# The characteristics of elevated blood pressure in abdominal obesity correspond to primary hypertension: a cross-sectional study

**DOI:** 10.1186/s12872-023-03150-w

**Published:** 2023-03-27

**Authors:** Jyrki Taurio, Elina J. Hautaniemi, Jenni K. Koskela, Arttu Eräranta, Mari Hämäläinen, Antti Tikkakoski, Jarkko A. Kettunen, Mika Kähönen, Onni Niemelä, Eeva Moilanen, Jukka Mustonen, Ilkka Pörsti

**Affiliations:** 1 Department of Internal Medicine, Tampere University Hospital, Tampere, Finland; 2 Faculty of Medicine and Health Technology, Tampere University, FI-33014 Tampere, Finland; 3 The Immunopharmacology Research group, Faculty of Medicine and Health Technology, Tampere University and Tampere University Hospital, Tampere, Finland; 4 Department of Clinical Physiology, Tampere University Hospital, Tampere, Finland; 5 Laboratory and Medical Research Unit, Seinäjoki Central Hospital, Seinäjoki, Finland

**Keywords:** Abdominal obesity, Hypertension, Hemodynamics, Impedance cardiography, Pulse wave analysis

## Abstract

**Background:**

Obesity-related hypertension and the associated metabolic abnormalities are considered as a distinct hypertensive phenotype. Here we examined how abdominal fat content, as judged by waist:height ratio, influenced blood pressure and hemodynamic profile in normotensive subjects and never-treated hypertensive patients.

**Methods:**

The 541 participants (20–72 years) underwent physical examination and laboratory analyses and were divided into age and sex-adjusted quartiles of waist:height ratio. Supine hemodynamics were recorded using whole-body impedance cardiography, combined with analyses of radial tonometric pulse wave form and heart rate variability.

**Results:**

Mean waist:height ratios in the quartiles were 0.46, 0.51, 0.55 and 0.62. Radial and aortic blood pressure, systemic vascular resistance, pulse wave velocity, markers of glucose and lipid metabolism, leptin levels and C-reactive protein were higher in quartile 4 when compared with quartiles 1 and 2 (p < 0.05 for all). Cardiac index was lower in quartile 4 versus quartile 1, while no differences were seen in heart rate variability, augmentation index, plasma renin activity, and aldosterone concentration between the quartiles. Linear regression analyses showed independent associations of abdominal obesity with higher aortic systolic and diastolic blood pressure, systemic vascular resistance, and pulse wave velocity (p < 0.05 for waist:height ratio in all regression models).

**Conclusion:**

Higher waist:height ratio was associated with elevated blood pressure, systemic vascular resistance, and arterial stiffness, but not with alterations in cardiac sympathovagal modulation or activation of the circulating renin-angiotensin-aldosterone system. Although obesity-related elevation of blood pressure has distinct phenotypic features, these results suggest that its main characteristics correspond those of primary hypertension.

**Trial registration:**

ClinicalTrails.gov NCT01742702 (date of registration 5th December 2012).

## Background

Obesity is a major global health risk, and the prevalence of obesity has doubled since 1980 [1]. In 2015, high body mass index (BMI) was estimated to account for 4 million deaths predominantly from cardiovascular diseases [1]. BMI has been the gold standard in the estimation of excess body fat, but it does not discriminate between fat and fat-free mass, which may lead to flawed results on body composition [2]. In 1996, Ashwell et al. reported that the correlation between tomography-measured intra-abdominal fat with BMI was 0.69, but for waist:height ratio (WHtR) the correlation was 0.83 (p < 0.001 for the difference). WHtR was concluded to be the better anthropometric predictor of intra-abdominal fat in both sexes [2]. High waist circumference that indicates excess abdominal fat content is also a predictor of future hypertension [3].

Excess visceral fat is associated with changes in glucose and lipid homeostasis that predispose to the development of hypertension [4]. Increased perivascular fat content and the associated changes in synthesis and release of adipokines may influence the recruitment of inflammatory cells in the vasculature, vascular smooth muscle proliferation, and the control of arterial tone [5]. Obesity related adipokines like leptin have been linked with several cardiovascular risk factors, but previous results about the association of leptin with arterial stiffness are inconsistent [6, 7]. Impaired endothelial function and increased arterial stiffness have been associated with obesity [8]. Elevated sympathovagal balance [9] and upregulation of the renin-angiotensin-aldosterone system (RAAS) [10] may also contribute to the cardiovascular changes in obesity. Obese subjects may have increased cardiac output, while systemic vascular resistance may be low in obese normotensives and normal or elevated in obese hypertensives [11].

Although the underlying mechanisms are not completely understood, elevated blood pressure (BP) in obese subjects is considered to have distinct phenotypic features [9, 12]. Obesity predisposes to impaired nocturnal BP dipping, increased prevalence of masked hypertension, higher exercise related increase in systolic BP, and treatment resistant hypertension [13]. However, when compared with essential hypertension, no definite instructions for the treatment of obesity-related hypertension in addition to weight reduction are included in the guidelines regarding e.g. the choices of antihypertensive medications [13].

To evaluate the hemodynamic features associated with abdominal obesity, we examined the influence of visceral fat content, defined as WHtR, on BP and hemodynamic profiles in normotensive and previously undiagnosed hypertensive subjects without antihypertensive medications. Cardiac autonomic modulation was evaluated utilizing analyses of heart rate variability (HRV).

## Methods

### Study subjects

The participants were recruited as previously described [14, 15]. All underwent physical examination and laboratory analyses for elevated BP [16]. Medical history and lifestyle habits including alcohol consumption and smoking were documented. Subjects with a history of coronary artery disease, stroke, cardiac failure or valve disease, heart rhythm other than sinus, chronic kidney disease, diabetes, secondary hypertension, alcohol or substance abuse, and psychiatric illnesses other than mild depression or anxiety were excluded.

Altogether 541 participants, aged 20–72 years, without antihypertensive medications were included. They were divided into age-adjusted quartiles of WHtR separately for sexes. The following medications were regularly used by the participants with no significant differences between the quartiles of WHtR: female hormones (contraception, hormone replacement therapy, n = 67), intrauterine hormonal device for contraception (n = 29), antidepressants (n = 25), vitamin-D supplements (n = 31), thyroxin (n = 16), inhaled glucocorticoids (n = 14), antihistamines (n = 12), statins (n = 11), proton pump inhibitors (n = 9), nonsteroidal anti-inflammatory agents (n = 4), anxiolytic agents (n = 4), allopurinol (n = 2), antiepileptics (n = 2), coxibs (n = 2), varenicline (n = 2), gabapentin or pregabalin (n = 2), warfarin (n = 1).

Signed informed consent was obtained from all participants. The study complies with the Declaration of Helsinki and was approved by the Ethics Committee of the Tampere University Hospital (study code R06086M) and the Finnish Medicines Agency (Eudra-CT registration number 2006-002065-39) and was registered in a database (ClinicalTrails.gov NCT01742702).

### Laboratory analyses

Blood and urine samples were drawn after ~ 12 h of fasting. Concentrations of leptin and adiponectin in plasma samples were determined using enzyme-linked immunosorbent assay (DuoSet ELISA; R&D Systems Europe Ltd, Abingdon, United Kingdom). Interassay coefficient of variation was 4.0% for leptin and 3.9% for adiponectin. The other laboratory analyses were performed as described previously in detail [15, 17].

### Experimental protocol

Hemodynamics were recorded as described previously [14, 15]. Electrodes for impedance cardiography placed on body surface, tonometric sensor on left radial pulsation, and oscillometric cuff to the right upper arm. The left arm was fixed to 90 degrees in a support. Hemodynamic data was captured continuously for 5 min, and mean values of each 1-minute period were calculated. The good repeatability and reproducibility of the measurements has been demonstrated [14, 15].

### Pulse wave analysis

As in our previous reports, radial pulse wave form was continuously captured using a tonometric sensor (Colin BP-508T, Colin Medical Instruments Corp., USA) [14, 16]. Aortic BP was derived with the SphygmoCor system (SpygmoCor PWMx, Atcor Medical, Australia) [18]. Aortic pulse pressure, augmentation index (AIx, augmented pressure/pulse pressure*100), AIx adjusted to heart rate 75/min (AIx@75), and central forward wave amplitude were determined [19]. Large arterial compliance was evaluated as the ratio of stroke volume to aortic pulse pressure [20].

### Whole-body impedance cardiography

Whole-body impedance cardiography (CircMon^R^, JR Medical Ltd., Tallinn, Estonia) was used to determine heart rate, stroke volume, cardiac output, pulse wave velocity (PWV), and extracellular water balance, as previously described [21–23]. Systemic vascular resistance was calculated from the tonometric BP and cardiac output by CircMon^R^. Stroke volume, cardiac output and systemic vascular resistance were related to body surface area and presented as indexes – SI, CI, and SVRI, respectively. The measured stroke volume and cardiac output values are in good agreement with 3-dimensional echocardiography [15] and the thermodilution and direct oxygen Fick methods [21], and the recorded PWV values show good correlation with ultrasound and tonometric values [22, 24].

### Frequency domain analysis of heart rate variability

Recorded electrocardiograms (sampling rate 200 Hz) were analyzed using Matlab (MathWorks Inc., Natick, Massachusetts, USA). Normal R-R intervals were recognized, and a beat was considered ectopic if the interval differed > 20% from previous values. Artefacts were processed using cubic spline interpolation method, and the frequency domain variables were calculated using Fast Fourier Transformation: (i) power in low frequency (LF) range (0.04–0.15 Hz), (ii) power in high frequency (HF) range (0.15–0.40 Hz), and (iii) LF/HF ratio [25].

### Statistics

Analysis of variance was applied for normally distributed variables and the Kruskal-Wallis and Mann-Whitney U-tests for non-normally distributed variables. The Bonferroni correction was applied in all post-hoc analyses. IBM SPSS Statistics Version 26 (IBM Corporation, Armonk, NY, USA) was used. The mean hemodynamic values from the minutes 3–5 were used when the signal was most stable. The LF power, HF power, and LF/HF ratio were transformed to natural logarithm for statistics due to skewed distributions, and the analyses were adjusted for heart rate [26]. The participants were divided to age-adjusted quartiles (Q) of WHtR separately for sexes.

Stepwise linear regression analyses were used to investigate factors associated with aortic systolic and diastolic BP, SVRI, and PWV. Smoking was categorized (current smokers, previous smokers, never smokers) using two discrete variables, alcohol consumption using three discrete variables (category either 0 or 1); cut-points for women 0, 1–7 (low), 8–14 (moderate), and ≥ 15 doses per week (high); for men 0, 1–14, 15–24, and ≥ 25 doses per week, respectively, according to the Finnish Guidelines [27]. The regression analyses included age, sex, WHtR, smoking status, categorized alcohol intake; plasma leptin, adiponectin, C-reactive protein (CRP), triglycerides, high-density lipoprotein (HDL) and low-density lipoprotein (LDL) cholesterol, sodium, calcium, parathyroid hormone (PTH), uric acid, renin activity, aldosterone; quantitative insulin sensitivity check index (QUICKI) [28] and estimated glomerular filtration rate (eGFR) as independent factors. In the analyses of aortic systolic and diastolic BP the model also included PWV, and in analyses of PWV the model also included mean aortic blood pressure. *P* < 0.05 was considered significant.

## Results

### Study population and laboratory values

Mean weight, waist circumference, and BMI were different in all quartiles of WHtR (Table 1). The difference in adiposity was substantial, as BMI was 9.0 (0.3) kg/m^2^ [mean (standard error)] higher in Q4 than in Q1. Mean BMI in the study participants was 26.8 (0.2) kg/m^2^. The Spearman correlation between WHtR and BMI was 0.895. Average height was slightly lower in Q4 of WHtR than in Q1 (Table 1).

**Table 1:** Study participants shown in age adjusted quartiles (Q) of waist:height ratio

Variable	Q1 (n = 132)	Q2 (n = 137)	Q3 (n = 138)	Q4 (n = 134)	Range (all quartiles)
Male / Female	68 / 64	71 / 66	71 / 67	68 / 66	
Age (years)	44.7 (12.4)	45.4 (11.6)	45.8 (11.0)	45.4 (11.4)	20–72
Waist:height ratio	0.46 (0.04)	0.51 (0.04)*	0.55 (0.04)*†	0.62 (0.05)*†‡	0.38–0.76
Weight (kg)	70.6 (12.7)	76.5 (13.6)*	82.1 (12.2)*†	94.1 (15.0)*†‡	45–135
Waist circumference (cm)	80.2 (9.6)	88.4 (10.1)*	95.6 (8.4)*†	106.5 (10.1)*†‡	63.0-132.0
Body mass index (kg/m^2^)	22.8 (2.5)	25.1 (2.5)*	27.4 (2.6)*†	31.9 (3.7)*†‡	17.9–42.1
Height (cm)	175.0 (10.0)	173.8 (9.6)	172.8 (8.2)	171.5 (9.0)*	150–203
Current smokers (%)	12	10	15	13	10–15
Alcohol (standard drinks/week)	2 [1–4]	2 [1–5]	2 [1–7]	3 [1–7]	0–42
Office systolic BP (mmHg)	134 (20)	137 (20)	142 (22)*	149 (20)*†	100–216
Office diastolic BP (mmHg)	85 (11)	86 (11)	90 (13)*†	95 (13)*†‡	54–135
Extracellular water balance	1.00 (0.09)	0.98 (0.10)	0.96 (0.09)*	0.97 (0.09)*	0.63–1.55

Results shown as mean (standard deviation) or median [25th -75th percentile]; *P < 0.05 vs. Q1; †*P <* 0.05 vs. Q2; ‡*P <* 0.05 vs. Q3

No differences were observed in the prevalence of smoking and average alcohol intake between the quartiles of WHtR. Office systolic BP was higher in Q3 than in Q1, and in Q4 than in Q2 and Q1 (Table 2). The difference in office systolic BP between Q4 vs. Q1 was 15 (3) mmHg. Office diastolic BP was highest in Q4, and higher in Q3 than in Q2 and Q1. The difference in office diastolic BP between Q4 vs. Q1 was 10 (1) mmHg. Extracellular water balance was lower in Q3 and Q4 than in Q1 (Table 1).

**Table 2:** Clinical characteristics and laboratory results in age adjusted quartiles (Q) of waist:height ratio

Variable	Q1	Q2	Q3	Q4	Range (all quartiles)
Hemoglobin (g/l)	142 (12)	144 (12)	146 (11)	145 (12)	113–177
Sodium (mmol/l)	140.7 (2.0)	140.2 (2.1)	140.3 (1.9)	140.3 (1.9)	134–146
Potassium (mmol/l)	3.82 (0.27)	3.75 (0.26)	3.83 (0.26)	3.85 (0.30)†	3.2–4.9
Calcium (mmol/l)	2.30 (0.11)	2.29 (0.10)	2.32 (0.12)	2.30 (0.10)	2.07–2.74
Parathyroid hormone (pmol/l)	4.4 (1.7)	4.3 (1.4)	4.5 (1.6)	5.0 (2.1)*†‡	1.4–9.9
C-reactive protein (mg/l)	0.6 [0.5-1.0]	0.6 [0.5–1.3]	1.0 [0.5–1.7]*	1.9 [0.8–3.1]*†‡	0.1–17.9
Uric acid (µmol/l)	277 (73)	287 (72)	313 (76)*†	328 (77)*†	103–600
Creatinine (µmol/l)	76 (14)	75 (14)	74 (13)	71 (14)*	42–116
Cystatin C (mg/l)	0.82 (0.14)	0.82 (0.16)	0.86 (0.14)*	0.87 (0.13)*†	0.47–1.31
Estimated GFR (ml/min/1.73m^2^)	102 (17)	102 (19)	97 (18)	96 (17)*	53–152
Leptin (ng/ml)	8.6 (8.5)	12.1 (9.2)	15.9 (12.8)*	28.0 (20.1)*†‡	0.2–92.3
Adiponectin (µg/ml)	4.3 (2.4)	3.9 (1.6)	3.4 (1.7)*	3.5 (1.7)*	0.5–18.2
Renin activity (ng Ang I/ml/h)	0.8 [0.5–1.3]	0.8 [0.4–1.3]	0.7 [0.4–1.3]	0.6 [0.4–1.1]	0.1–10.0
Aldosterone (pmol/l)	436 [306–636]	434 [329–581]	459 [338–595]	416 [304–539]	68-1704
Aldosterone:renin ratio	597 [398–791]	634 [383–884]	640 [430–948]	632 [389–979]	45-2701
Fasting plasma					
Total cholesterol (mmol/l)	4.9 (1.0)	4.9 (1.0)	5.3 (1.1)*†	5.4 (1.0)*†	2.5-9.0
Triglycerides (mmol/l)	0.9 [0.6–1.2]	1.0 [0.7–1.3]	1.1 [0.8–1.5]*	1.2 [1.0–2.0]*†	0.3–5.5
HDL cholesterol (mmol/l)	1.8 (0.5)	1.6 (0.4)*	1.5 (0.4)*	1.4 (0.4)*†	0.7–3.1
LDL cholesterol (mmol/l)	2.8 (0.9)	2.8 (0.9)	3.2 (0.9)*†	3.3 (0.9)*†	0.8–5.8
Insulin (mU/l)	6.0 (3.6)	6.9 (4.7)	8.2 (5.3)*	11.5 (7.9) *†‡	1.0-51.8
Glucose (mmol/l)	5.3 (0.5)	5.4 (0.5)	5.5 (0.5)*	5.7 (0.8)*†‡	4.1–7.5
QUICKI	0.380 (0.056)	0.366 (0.036)*	0.354 (0.032)*	0.338 (0.033)*†‡	0.268–0.740

Results shown as mean (standard deviation) or median [25th -75th percentile]; BP, blood pressure; estimated GFR, estimated glomerular filtration rate based on cystatin C (CKD-EPI) [45]; QUICKI, quantitative insulin sensitivity check index [28]; *P < 0.05 vs. Q1; †*P <* 0.05 vs. Q2; ‡*P <* 0.05 vs. Q3

Average blood hemoglobin and plasma concentrations of sodium, potassium, and calcium were within the normal range in all quartiles (Table 2). Plasma PTH and CRP were highest in Q4, while uric acid was higher in Q3 and Q4 than in Q1 and Q2. Creatinine and cystatin C concentrations presented with minor differences between the quartiles, while eGFR derived from cystatin C was slightly lower in Q4 than in Q1. Less than 4% of the subjects presented with values of the above variables that were outside the normal range (Table 2).

Plasma leptin concentration was clearly highest in Q4, and higher in Q3 than in Q1, while adiponectin was lower in Q3 and Q4 than in Q1. Plasma renin activity, aldosterone concentration, and aldosterone:renin ratio did not differ between the quartiles (Table 2).

Q3 and Q4 of WHtR had less favorable lipid profiles than Q1 and Q2. Plasma total cholesterol, LDL cholesterol, and triglycerides were above the normal range in 53%, 47%, and 15% of the subjects, respectively. Plasma HDL cholesterol was below the normal range in 9% of the subjects. Fasting plasma insulin and glucose were higher in Q3 than in Q1, while both insulin and glucose were highest in Q4. Insulin was above the normal range in 2% and glucose in 13% of the participants. Based on QUICKI, insulin sensitivity was highest in Q1 and lowest in Q4 (Table 2).

### Blood pressure, arterial stiffness, cardiac variables, and heart rate variability

**Fig. 1:**
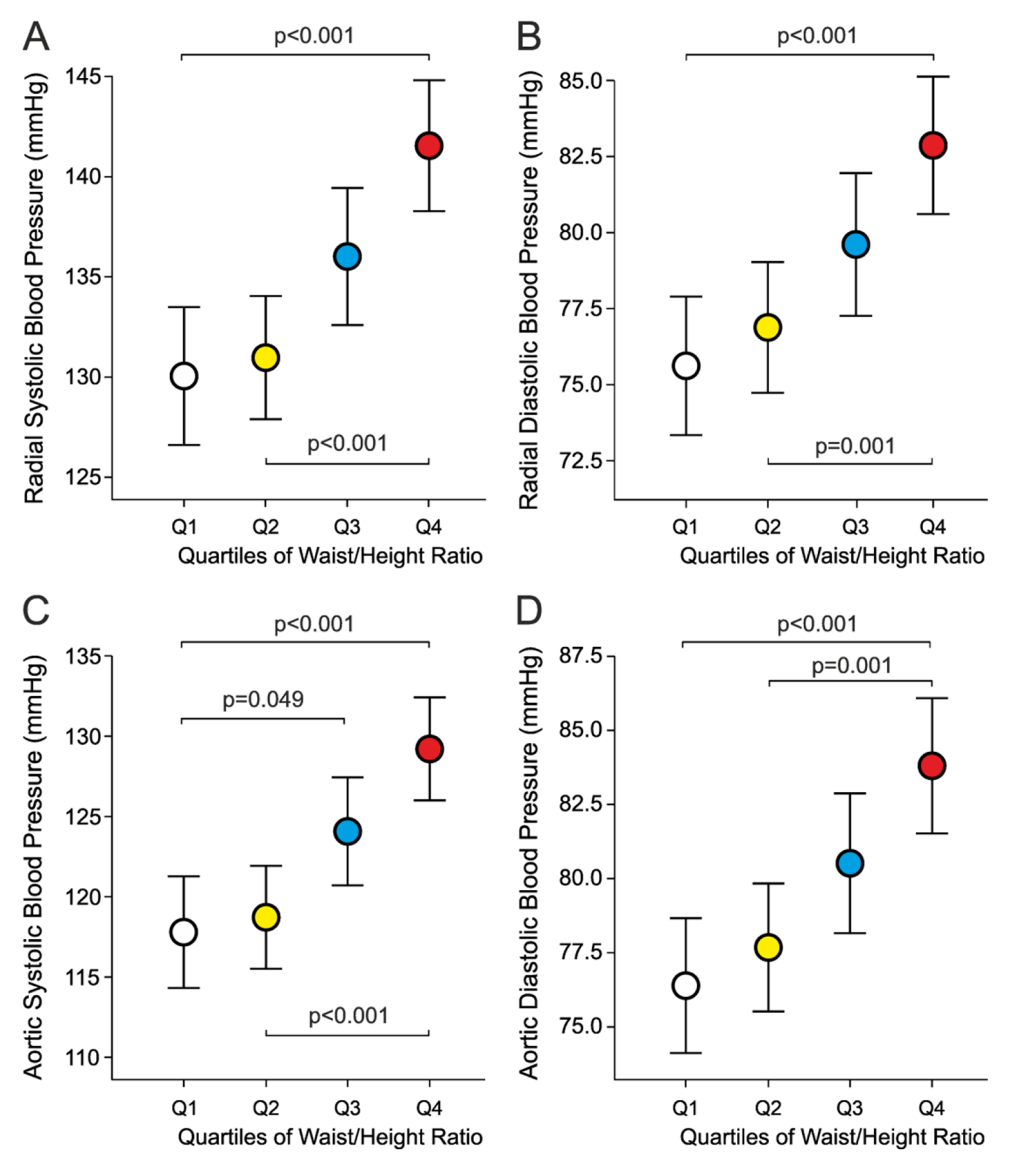
Radial systolic (A) and diastolic (B) blood pressure, and aortic systolic (C) and diastolic (D) blood pressure during laboratory measurements in 541 subjects divided separately for sexes into age-adjusted quartiles of waist/height ratio; mean ± confidence interval of the mean; significant differences shown between groups (*P* < 0.05)

Radial systolic and diastolic BP were elevated in Q4 when compared with Q1 and Q2 (Fig. 1A and B). Aortic systolic BP was higher in Q3 when compared with Q1, and aortic systolic and diastolic BP were higher in Q4 than in Q1 and Q2 (Fig. 1 C and 1D). The difference in aortic systolic / diastolic BP between Q4 vs. Q1 was 11 (2) / 7 (2) mmHg.

**Fig. 2:**
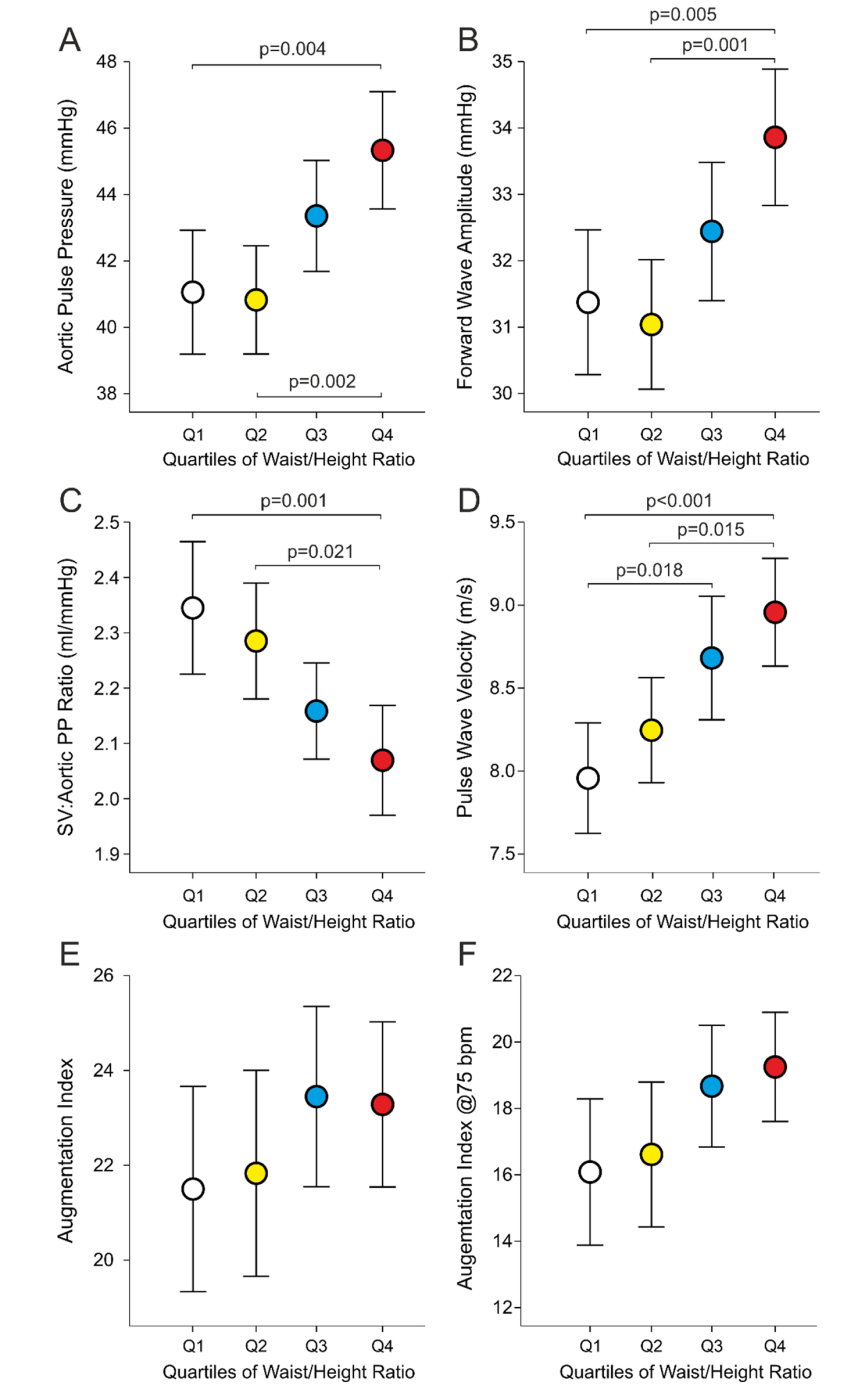
Aortic pulse pressure (A), forward wave amplitude (B), stroke volume to aortic pulse pressure ratio (C), pulse wave velocity (D), augmentation index (E), and augmentation index adjusted to heart rate of 75 beats per minute (bpm) (F); statistics as in Fig. 1

Aortic pulse pressure and forward wave amplitude were higher in Q4 than in Q1 and Q2 (Fig. 2A and B). Evaluated aortic compliance (stroke volume to central pulse pressure ratio) was lower in Q4 than in Q1 and Q2 (Fig. 2C), while aortic to popliteal PWV was higher in Q4 than in Q1 and Q2, and in Q3 than in Q1 (Fig. 2D). The difference in PWV between Q4 vs. Q1 was 1.0 (0.2) m/s. No differences were observed in AIx or AIx@75 (Fig. 2E F).

**Fig. 3:**
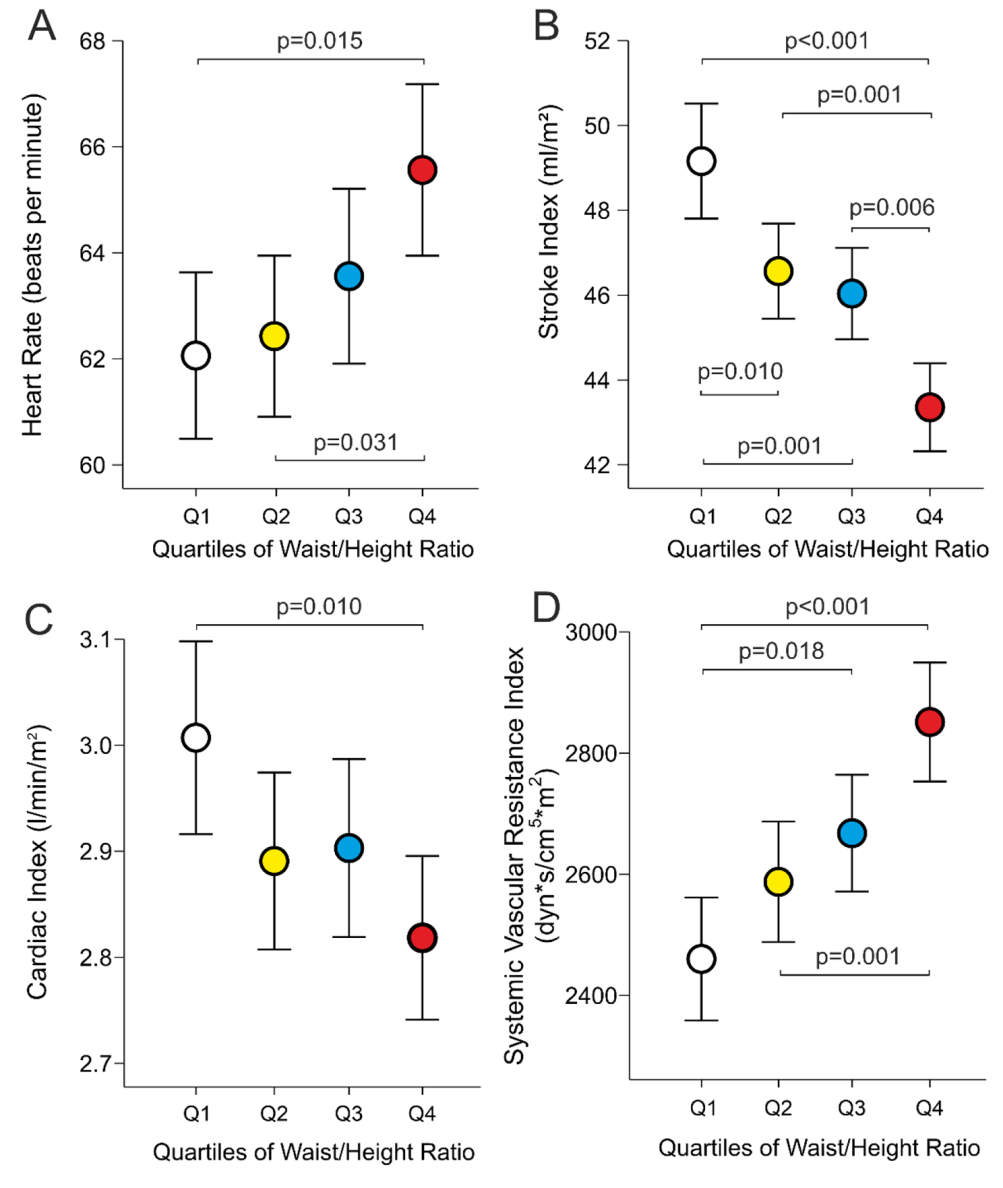
Heart rate (A), stroke index (B), cardiac index (C), systemic vascular resistance index (D) in 541 subjects; statistics as in Fig. 1

Heart rate was higher in Q4 than in Q1 and Q2 (Fig. 3A), while SI was different in all other quartiles but not between Q2 and Q3 (Fig. 3B). CI was lower in Q4 than Q1 (Fig. 3C), while SVRI was higher in Q4 than in Q1 and Q2, and in Q3 than in Q1. The difference in SVRI between Q4 vs. Q1 was 391 (71) dyn*s/cm^5^*m^2^ (Fig. 3D).

**Fig. 4:**
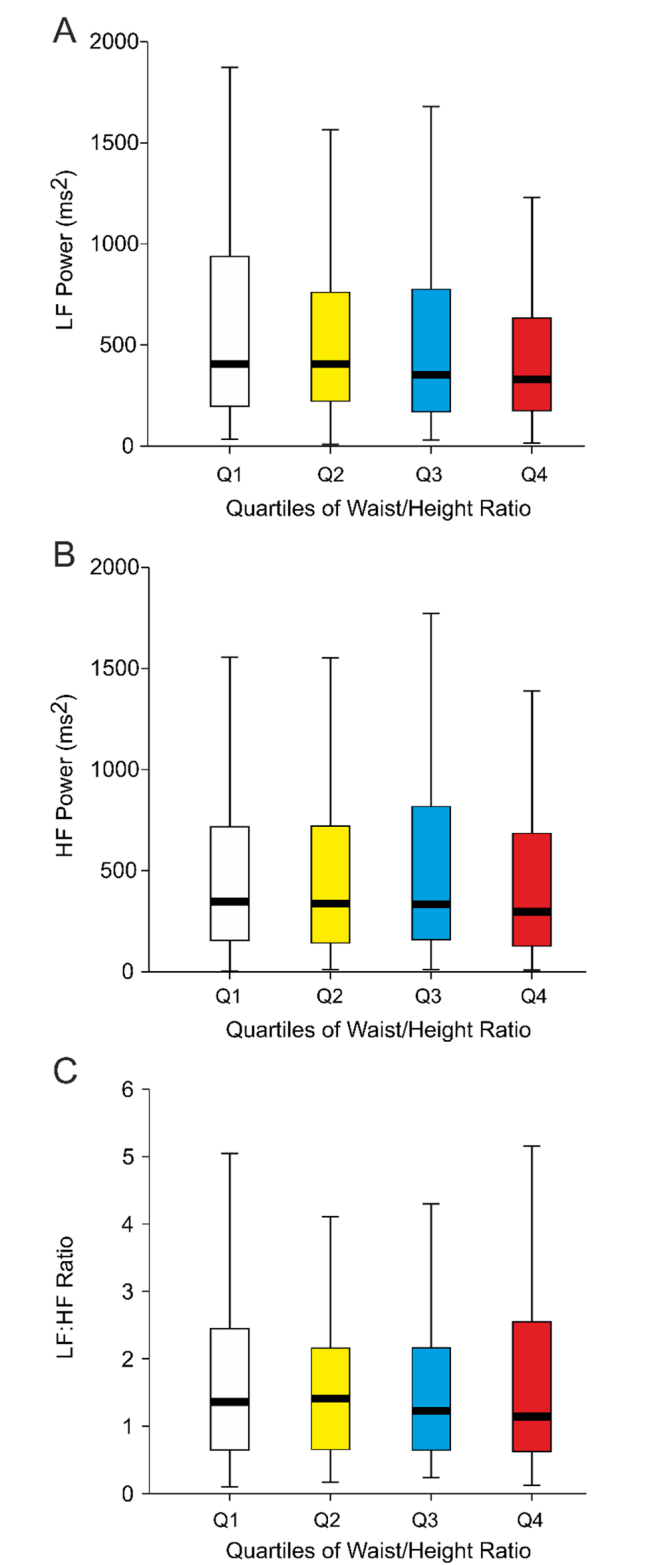
Box plots show heart rate variability in age-adjusted quartiles of waist/height ratio divided separately for sexes. Low frequency (LF) power (A), high frequency (HF) power (B), and LF/HF ratio (C); median (thick line inside box), 25th -75th percentile (box), range (whiskers); outliers were omitted from the figures but were included in the statistics with logarithmically transformed values

The HRV measurements consisted of LF and HF power and LF/HF ratio determinations (Fig. 4). No differences were observed between the quartiles in these analyses.

### Regression analyses of hemodynamic variables

The linear regression analyses showed statistically significant independent associations of (1) WHtR, PWV, eGFR, QUICKI, LDL cholesterol and triglycerides with aortic systolic and diastolic BP; (2) plasma sodium concentration and age with aortic systolic BP; and (3) male sex and high alcohol consumption category with aortic diastolic BP (Table 3).

**Table 3:** Linear regression analyses with stepwise elimination of explanatory factors for aortic blood pressure, systemic vascular resistance index, and pulse wave velocity

**Aortic systolic BP (mmHg) R** ^**2**^ ** = 0.398**	**Unstandardized coefficient B**	**Standardized coefficient Beta**	**P value**
(constant)	-4.147		0.935
Pulse wave velocity	2.218	0.225	< 0.001
eGFR	-0.219	-0.198	< 0.001
LDL cholesterol	3.469	0.165	< 0.001
Waist:height ratio	32.486	0.119	0.006
Age	0.201	0.117	0.016
Plasma sodium	0.785	0.078	0.024
QUICKI	-42.355	-0.092	0.016
Triglycerides	-1.934	-0.092	0.019
**Aortic diastolic BP (mmHg) R** ^**2**^ ** = 0.346**	**Unstandardized coefficient B**	**Standardized coefficient Beta**	**P value**
(constant)	92.850		< 0.001
eGFR	-0.200	-0.266	< 0.001
Pulse wave velocity	1.276	0.190	< 0.001
LDL cholesterol	2.334	0.163	< 0.001
QUICKI	-36.109	-0.115	0.003
Male sex	3.071	0.113	0.002
Triglycerides	-1.828	-0.127	0.003
High alcohol consumption category	8.757	0.091	0.014
Waist:height ratio	17.861	0.096	0.031
SVRI (dyn*s/cm ^**5**^ *m ^**2**^ **) R** ^**2**^ ** = 0.219**	**Unstandardized coefficient B**	**Standardized coefficient Beta**	**P value**
(constant)	1659		< 0.001
Waist:height ratio	1911	0.235	< 0.001
eGFR	-4.981	-0.151	0.002
LDL cholesterol	77.992	0.125	0.006
Present smoking	-178.938	-0.099	0.011
Age	5.297	0.103	0.037
**Pulse wave velocity (m/s) R** ^**2**^ ** = 0.553**	**Unstandardized coefficient B**	**Standardized coefficient Beta**	**P value**
(constant)	-0.736		0.127
Age	0.074	0.475	< 0.001
Uric acid	0.005	0.196	< 0.001
Mean aortic pressure	0.018	0.152	< 0.001
Waist:height ratio	4.895	0.199	< 0.001
Leptin	-0.013	-0.113	0.001
Aldosterone	3.86 × 10^− 4^	0.081	0.008
Present smoking	-0.479	-0.087	0.003
Triglycerides	0.135	0.071	0.031

See Methods for included variables; BP, blood pressure; eGFR, estimated glomerular filtration rate using the CKD-EPI creatinine-cystatin C equation; HDL, high density lipoprotein; LDL, low density lipoprotein; QUICKI, quantitative insulin sensitivity check index; SVRI, systemic vascular resistance index; CRP, C-reactive protein

WHtR, eGFR, LDL cholesterol, present smoking, and age were independently associated with SVRI. Age, uric acid, mean aortic pressure, WHtR, leptin, aldosterone, present smoking, and triglycerides were independently related with PWV (Table 3).

## Discussion

Obesity-related hypertension is considered as a distinct phenotype [9, 12], but the underlying mechanisms remain elusive. The pathophysiology of obesity-induced hypertension involves various potential pathways [4, 5, 8–12]. Excess visceral adiposity is associated with altered secretion of bioactive peptides like adiponectin, leptin, interleukin-6, and tumor necrosis factor-α, predisposing to inappropriate inflammatory responses, insulin resistance, increased sympathetic activity and RAAS activation. These changes impair endothelial function and increase tubular reabsorption of sodium and water, leading to elevated BP [4, 9]. In the present study, aortic systolic BP, systemic vascular resistance, and large arterial stiffness were elevated with higher intra-abdominal fat content in the absence of changes in volume balance, modulation of cardiac sympathovagal balance, or circulating RAAS. The differences between the study quartiles emphasized the influences of SVRI and arterial stiffness as BP-elevating factors related with higher WHtR, and the regression analyses confirmed that WHtR was independently associated with aortic BP, SVRI, and large arterial stiffness.

WHtR better correlates with intra-abdominal fat content than waist circumference, BMI, or waist to hip ratio [2]. WHtR also presents with stronger inverse correlation with cardiovascular health than waist circumference [29]. A cutoff value of 0.5 for WHtR has been suggested for the risk assessment of cardiovascular disease [30], and this value was exceeded in three of the present quartiles that exhibited mean BMI values ranging from 25.1 to 31.9 kg/m^2^. According to a recent survey, mean BMI in Finland is 27.7 kg/m^2^ in men and 27.5 kg/m^2^ in women, while 27% of men and 26% of women aged 30–64 years are obese [31]. The present study cohort with a mean BMI of 26.8 kg/m^2^ well corresponds to the concurrent Finnish population.

Previously, a direct correlation between BMI and plasma aldosterone concentration was reported in overweight patients independent of age, sex and sodium intake [32]. In addition, weight loss was found to reduce plasma renin activity and aldosterone concentration in overweight subjects [33]. In the present study, no differences were detected in plasma renin activity, aldosterone concentration, or aldosterone:renin ratio between the quartiles, and measurements of extracellular volume balance did not indicate volume retention with higher WHtR. Thus, there were no findings indicating changes in circulating RAAS activity between the WHtR quartiles. However, increased WHtR was associated with an unfavorable lipid profile, and in linear regression analyses LDL cholesterol was associated with systolic and diastolic BP and SVRI, as previously reported [34].

As expected, systolic and diastolic BP were increased with higher WHtR. Heart rate was also increased, but stroke volume and cardiac output adjusted to body surface area were decreased with higher WHtR, suggesting that hyperdynamic circulation was not the cause for elevated BP. In contrast, SVRI was clearly increased with higher WHtR. Like in essential hypertension [35], the mechanisms leading to elevated SVRI are probably multifactorial. The hemodynamic pattern of reduced CI and increased SVRI has been shown in subjects with essential hypertension [36]. Also, Krzesiński et al. reported that hypertensive patients with or without abdominal obesity presented with similar SVRI, whereas left ventricular contractility and thoracic fluid content were lower in hypertensive subjects with abdominal obesity [37].

PWV is an acknowledged measure of large arterial stiffness [38]. We found that PWV, and also forward wave amplitude that has been associated with aortic stiffness [19], were increased with higher fat content in the central body. However, the indices of wave reflection AIx and AIx@75 [24, 38], did not differ between the quartiles. AIx is influenced by arterial stiffness, but also by height, sex, ventricular ejection duration, heart rate, and systemic vascular resistance [24, 38]. We also evaluated arterial compliance by calculating the ratio of stroke volume to aortic pulse pressure [20], and found that this variable was lower with higher WHtR. Our findings strongly support the view that obesity is associated with increased arterial stiffness [39].

Obesity related increase in plasma leptin concentration is assumed to induce unfavorable cardiovascular changes via the activation of the sympathetic nervous system [40] and RAAS [41], leading to hypertension and increased large arterial stiffness. Studies investigating the association of leptin and arterial stiffness have provided variable results [6, 7]. In the present study, subjects with high WHtR presented with elevated plasma leptin levels, and a 3.3-fold difference in leptin was detected between the highest and lowest quartiles of WHtR. Leptin was moderately related with PWV in the regression analysis, but RAAS activity or sympathetic modulation of HRV were not increased. Thus, the association of WHtR with BP and arterial stiffness may be more related to abdominal obesity itself than high level of circulating leptin.

Excess body fat has been associated with increased sympathetic activity, but the matter remains controversial [42–44]. We evaluated modulation of cardiac autonomic tone using HRV analyses, and found no differences in LF power or HF power, reflecting predominantly sympathetic and parasympathetic influences, respectively [25], or LF/HF ratio between the quartiles of WHtR. Previously, Skrapari et al. reported lower LF and HF power in obese (BMI ~ 40 kg/m^2^) versus lean (BMI ~ 22 kg/m^2^) subjects [43], while Hillebrand et al. found that BMI was associated with LF power but not with HF power [44]. Emdin et al. reported decreased LF power throughout the 24-hour recording period in obese (BMI ~ 35 kg/m^2^) versus lean (BMI ~ 24 kg/m^2^) subjects, while HF power was lower, and the LF/HF ratio was higher, during the postprandial phases [42]. Higher daytime LF/HF ratios have been related with higher plasma insulin concentrations independent of BMI, sex, age, and heart rate [42]. In the present study, subjects in the upper quartiles of WHtR were more insulin resistant based on their QUICKI values, but no parallel changes in cardiac sympathovagal modulation were observed.

### Study Limitations

Non-invasive recordings of hemodynamics were utilized in this study, which can be considered a limitation. Stroke volume and cardiac output were evaluated from the bioimpedance signal based on a mathematical algorithm and simplification of physiology [21]. However, these methods have been validated against invasive measurements, 3-dimensional echocardiography recordings, and carotid-femoral measurements of PWV [15, 18, 21, 24]. Although this study presented associations between BP, systemic vascular resistance, HRV, and arterial stiffness, the cross-sectional design does not allow conclusions about causality. Importantly, the participants included in this study were without antihypertensive medications that can cause significant confounding during hemodynamic measurements.

## Conclusion

The present results showed that elevated BP related to abdominal obesity was characterized by increased systemic vascular resistance and arterial stiffness, but not by increased cardiac sympathovagal modulation, volume retention, or activation of the circulating RAAS. Although high BP in obese subjects has been characterized by distinct phenotypic features including increased sympathetic tone, impaired endothelium-mediated vasodilatation and RAAS upregulation [9, 12], the present results suggest that the most characteristic features related with elevated BP during higher WHtR are corresponding to those in primary hypertension. Prospective future studies are required to evaluate the clinical significance of the principal phenotypic features related with abdominal obesity.

## Data Availability

The datasets of the current study are available from the corresponding author on reasonable request.

## Abbreviations

AIx: augmentation index
AIx@75: augmentation index adjusted to heart rate 75/min
BMI: body mass index
BP: blood pressure
CI: cardiac index
CRP: C-reactive protein
eGFR: estimated glomerular filtration rate
HDL: high-density lipoprotein
HF: power in high frequency range
HRV: heart rate variability
LDL: low-density lipoprotein
LF: power in low frequency range
PTH: parathyroid hormone
PWV: pulse wave velocity
QUICKI: quantitative insulin sensitivity check index
RAAS: renin-angiotensin-aldosterone system
SI: stroke index
SVRI: systemic vascular resistance index
WHtR: waist:height ratio
